# Peat Bog Wildfire Smoke Exposure in Rural North Carolina Is Associated with Cardiopulmonary Emergency Department Visits Assessed through Syndromic Surveillance

**DOI:** 10.1289/ehp.1003206

**Published:** 2011-10-01

**Authors:** Ana G. Rappold, Susan L. Stone, Wayne E. Cascio, Lucas M. Neas, Vasu J. Kilaru, Martha Sue Carraway, James J. Szykman, Amy Ising, William E. Cleve, John T. Meredith, Heather Vaughan-Batten, Lana Deyneka, Robert B. Devlin

**Affiliations:** 1 Environmental Public Health Division, National Health and Environmental Effects Research Laboratory, and; 2 National Exposure Research Laboratory, U.S. Environmental Protection Agency, Research Triangle Park, North Carolina, USA; 3 Environmental Sciences Division, National Exposure Research Laboratory, U.S. Environmental Protection Agency, NASA Langley Research Center, Hampton, Virginia, USA; 4 Department of Emergency Medicine, School of Medicine, University of North Carolina, Chapel Hill, North Carolina, USA; 5 Pitt County Memorial Hospital, Greenville, North Carolina, USA; 6 Brody School of Medicine at East Carolina University, Department of Cardiovascular Sciences and the East Carolina Heart Institute, Greenville, North Carolina, USA; 7 North Carolina Division of Public Health, North Carolina Department of Health and Human Services, Raleigh, North Carolina, USA

**Keywords:** cardiopulmonary health effects, satellite data, syndromic surveillance, wildfire smoke exposure

## Abstract

Background: In June 2008, burning peat deposits produced haze and air pollution far in excess of National Ambient Air Quality Standards, encroaching on rural communities of eastern North Carolina. Although the association of mortality and morbidity with exposure to urban air pollution is well established, the health effects associated with exposure to wildfire emissions are less well understood.

Objective: We investigated the effects of exposure on cardiorespiratory outcomes in the population affected by the fire.

Methods: We performed a population-based study using emergency department (ED) visits reported through the syndromic surveillance program NC DETECT (North Carolina Disease Event Tracking and Epidemiologic Collection Tool). We used aerosol optical depth measured by a satellite to determine a high-exposure window and distinguish counties most impacted by the dense smoke plume from surrounding referent counties. Poisson log-linear regression with a 5-day distributed lag was used to estimate changes in the cumulative relative risk (RR).

Results: In the exposed counties, significant increases in cumulative RR for asthma [1.65 (95% confidence interval, 1.25–2.1)], chronic obstructive pulmonary disease [1.73 (1.06–2.83)], and pneumonia and acute bronchitis [1.59 (1.07–2.34)] were observed. ED visits associated with cardiopulmonary symptoms [1.23 (1.06–1.43)] and heart failure [1.37 (1.01–1.85)] were also significantly increased.

Conclusions: Satellite data and syndromic surveillance were combined to assess the health impacts of wildfire smoke in rural counties with sparse air-quality monitoring. This is the first study to demonstrate both respiratory and cardiac effects after brief exposure to peat wildfire smoke.

On 1 June 2008, a lightning strike initiated a fire in the eastern plains of North Carolina. Low humidity and prolonged drought contributed to the spread of the fire into the Pocosin Lakes National Wildlife Refuge, where it smoldered through rich deposits of peat. Peat in the refuge was, on average, 3 ft and in places up to 15 ft deep. Poor oxygen supply during combustion of carbon in the decomposing vegetation produced massive amounts of smoke. The region exposed to the hazardous levels of air pollution generated by the fire was largely rural, sparsely populated, and economically disadvantaged.

To investigate health effects associated with this fire, we obtained data on daily emergency department (ED) visits for cardiac and respiratory conditions for eastern North Carolina counties reported through the statewide syndromic surveillance system. Satellite measurements of aerosol optical depth (AOD) were used to determine a 3-day window of dense plume. We estimated relative risks (RRs) associated with these days and 5-lag days for the counties most impacted by the smoke plume and the surrounding, less impacted, referent counties.

Air pollution is a complex mixture of gases and particles whose toxicity depends on the type of fuel and conditions of combustion. There are > 100 epidemiological studies demonstrating mortality and morbidity associated with both chronic and acute exposures to air pollution, mostly focused on emissions of burning fossil fuels in automobiles, diesel engines, and coal- or oil-fired power plants [U.S. Environmental Protection Agency (EPA) 2009b]. During episodes of wildfires, affected regions experience acute exposures, with concentrations that are orders of magnitude larger than observed in urban centers. However, health implications of the exposure to wildfire emissions are less well understood (Naeher et al. 2007). In contrast to health studies of urban air pollution, wildfire studies are constrained by the size and distribution of the exposed population, duration of the episode, and the retrospective accessibility of data on exposure and health outcomes. The most comprehensive studies, conducted for forest fires near large metropolitan areas, have reported significant increases in symptoms and exacerbations of underlying respiratory illnesses (Delfino et al. 2009; Duclos et al. 1990; Johnson et al. 2005; Künzli et al. 2006). Less-conclusive results have been found within smaller cohort and convenience sample–based studies and less-populated regions (Moore et al. 2006; Mott et al. 2005). The effects of exposure on cardiovascular outcomes have been reported with more varied results (Delfino et al. 2009; Moore et al. 2006; Mott et al. 2005; Sastry 2002; Vedal and Dutton 2006), in part due to lack of statistical power.

Climate-related changes and past land-use practices are expected to increase the risk of wildfires in upcoming decades (National Interagency Fire Center 2009). In addition to the forests, of particular concern is the vulnerability of large deposits of peat bogs to wildfires. Peat soil accounts for approximately 2% of global land cover, mostly in the boreal regions, where it has been traditionally harvested for energy. In drought conditions, peat bogs are exceptionally susceptible to ignition, can smolder indefinitely, and are notoriously difficult to extinguish. Although less common than forest fires, they have an important impact on regional climate and ecosystems. In 1997, Indonesian peat fires released the equivalent of 13–40% of the mean annual global carbon emissions from fossil fuels and contributed to the largest annual increase in atmospheric carbon dioxide (CO_2_) in four decades (Page et al. 2002). Despite the large impact of peat fires on the environment, significantly less is known about the associated health effects. One study of the Indonesian fires reported > 500 haze-related deaths, 290,000 cases of asthma exacerbation, 58,000 cases of bronchitis, and 1,440,000 cases of acute respiratory infection between September and November 1997 (Kunii et al. 2002).

## Methods

We obtained daily counts of ED visits from the North Carolina Disease Event Tracking and Epidemiologic Collection Tool (NC DETECT 2010), a statewide, early-event detection and public health surveillance system that records daily ED visits from 111 of 114 civilian North Carolina EDs. We considered visits for selected cardiovascular and respiratory outcomes by adults throughout the eastern portion of the state, as well as county of residence, sex, age, date of admission, and discharge ICD-9-CM [*International Statistical Classification of Diseases and Related Health Problems, Ninth Revision, Clinical Modification* (U.S. Public Health Service and Health Care Financing Administration 1980)] codes for all visits. Prior to the analysis, outcomes of interest were defined through ICD-9-CM codes for asthma (493), chronic obstructive pulmonary disease (COPD) (491–492), pneumonia and acute bronchitis (481, 482, 485, 486, and 466), upper respiratory tract infections (URIs) (465), heart failure (428), cardiac dysrhythmia (427), and myocardial infarction (410, 411.1). ICD-9-CM codes grouped into all respiratory and all cardiac outcomes are listed in Table 1. Cardiopulmonary symptoms (ICD-9-CM code 786) were grouped separately. For respiratory-related outcomes, the population was stratified into two age groups: 19–64 years and ≥ 65 years. The small numbers of ED visits for respiratory-related events in those < 19 years of age precluded analysis of this subgroup. Cardiac outcomes were also stratified into two age groups: 45–64 years and ≥ 65 years. There were not enough visits for cardiovascular events for people < 45 years of age to be informative. The population was dichotomized to differentiate causes of cardiovascular outcomes associated with ischemic heart disease and left ventricular dysfunction, which are less prevalent in the younger population. The human subjects institutional review boards of the University of North Carolina at Chapel Hill, East Carolina University, and the U.S. Environmental Protection Agency approved the study.

**Table 1:** Total counts of ED visits in the exposed and referent counties by outcomes, age group, and sex, between 1 June and 14 July 2008.

Age (years)			Sex		
Counties/outcomes	Total	< 65	≥ 65	Female	Male
Exposed counties									
Respiratory outcomes									
All*a*	4,702		3,485		1,217		2,963		1,739
Asthma (ICD-9 493)	2,081		1,775		306		1,463		618
COPD (ICD-9 491, 492)	647		314		333		317		330
Pneumonia and acute bronchitis (ICD-9 481, 482, 485, 486, 466)	1,053		607		446		575		478
URIs (ICD-9 465)	444		189		255		202		242
Cardiac outcomes									
All*b*	6,078		2,037		4,041		3,357		2,721
Myocardial infarction (ICD-9 410, 411)	444		189		255		202		242
Heart failure (ICD-9 428)	1,817		579		1,238		1,068		749
Cardiac dysrhythmias (ICD-9 427)	1,756		538		1,218		937		819
Respiratory/other chest symptoms (786)	7,716		5,752		1,964		4,532		3,184
Referent counties									
Respiratory outcomes									
All*a*	6,074		4,347		1,727		3,819		2,255
Asthma (ICD-9 493)	2,199		1,886		313		1,591		608
COPD (ICD-9 491, 492)	1,158		558		600		601		557
Pneumonia and acute bronchitis (ICD-9 481, 482, 485, 486, 466)	1,815		1,146		669		1,039		777
URIs (ICD-9 465)	490		429		61		344		146
Cardiac outcomes									
All*b*	7,999		2,704		5,295		4,279		3,720
Myocardial infarction (ICD-9 410, 411)	674		334		340		288		386
Heart failure (ICD-9 428)	2,374		740		1,634		1,337		1,037
Cardiac dysrhythmias (ICD-9 427)	2,580		785		1,795		1,381		1,199
Respiratory/other chest symptoms (786)	10,102		7,801		2,301		5,968		4,134
Minimum ages for respiratory and cardiac outcomes were 19 and 45 years, respectively.**a**All respiratory ICD-9 codes: 465, 466, 480, 481, 482, 483, 484, 485, 486, 490, 491, 492, 493. **b**All cardiac ICD-9 codes: 410, 411, 413, 415, 416, 417, 420, 421, 422, 423, 424, 425, 426, 427, 428, 429, 434, 435, 444, 445, 451.									

**Figure 1:**
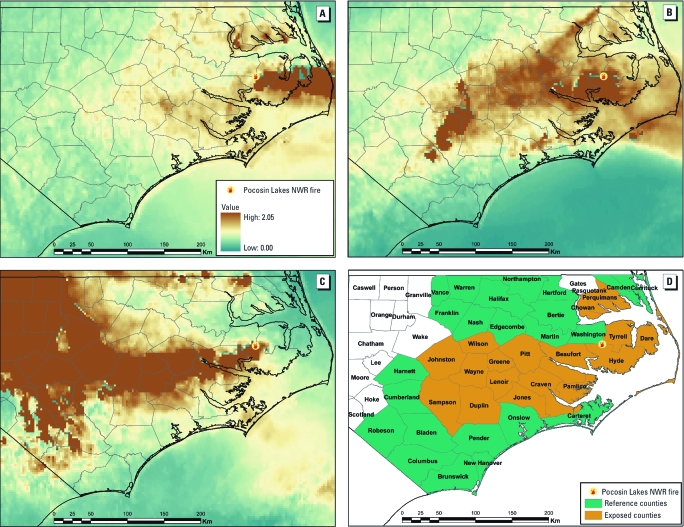
Aerial maps showing counties impacted by the Evans Road Fire at the Pocosin Lakes National Wildlife Refuge on 10, 11, and 12 June 2008 (*A*, *B*, and *C*, respectively) as measured by satellite AOD images. (*D*) Assignment of counties as exposed or referent.

**Figure 2:**
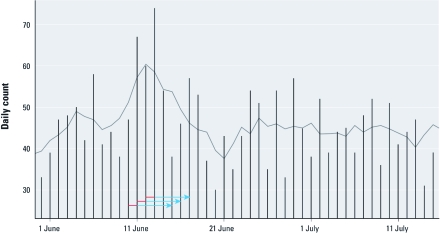
Daily counts of asthma-related ED visits in the exposed counties. Arrows represent the 3 days of high exposure (red) and the subsequent 5 lag days (blue).

The study period was defined as the time between the onset of the wildfire through mid-July when controlled flooding, increased humidity, and the first rainfall largely contained the fire (1 June–14 July 2008). During this period, average daily temperatures ranged from 69 to 86^o^F, with overnight lows always < 75^o^F [see Supplemental Material, Figure 1 (http://dx.doi.org/10.1289/ehp.1003206)]. For most of the period, winds blew from the west, and smoke affected only a few sparsely populated neighboring counties. However, on 10 June, easterly winds directed the smoke plume inland, exposing a large portion of eastern and central North Carolina for a 3-day period (Figure 1A–C). On 12 June, the maximum 1-hr concentration of fine particulate matter with diameter ≤ 2.5 µm (PM_2.5_) exceeded 200 μg/m^3^ at ground-based monitors located 200 km from the fire. We defined 10–12 June as a window of high exposure, and estimated risks associated with the exposure days and a 1- to 5-day lag period after each exposure day relative to the nonexposed days of the 6-week study period. Figure 2 illustrates the window of exposure and lagged days of exposure along with the daily asthma visits. The RRs for lag days 0–5 were summarized to define cumulative risk of exposure.

County-level exposure to the plume was classified using AOD, measured by instruments aboard a geostationary operational environmental satellite. AOD is a unitless measure with scale from 0 to 2 of the atmospheric scattering and absorption of light by aerosols, where larger values indicate higher concentration of particles in the atmosphere and lower visibility. Half-hour, 4 × 4 km resolution grid maps of AOD were averaged over the available daytime hours. AOD has been shown to be a good surrogate for boundary layer fine particulate matter concentrations and a predictor of the Air Quality Index, a nationally uniform index for reporting daily air quality (Al-Saadi et al. 2005; Engel-Cox et al. 2004; U.S. EPA 2009a; Wang and Christopher 2003). Typical background levels of AOD for this region were well below 0.5. Based on the sharp difference between the high-density plume and background, we chose an AOD of ≥ 1.25 as an indicator of the high-density plume. Counties with a minimum of 25% of the geographic area exceeding this threshold were defined as exposed to the smoke plume for each day in the high-exposure window. The operational algorithm of the satellite considers high AOD values created by strong reflectance from clouds as unreliable and removes them from the standard data product. At times, the dense smoke plume was classified as a cloud, resulting in missing AOD values on the interior of the plume. We considered such values as right-censored and classified the respective grid cells as exposed to the plume.

The study population resided in 42 contiguous counties in eastern North Carolina. One sparsely populated county (Gates County) was significantly impacted by another wildfire and was excluded from this analysis. Counties with smoke exposure on at least 2 days were considered exposed (18 counties in Figure 1D). The 23 referent counties were exposed 1 day (15 counties) or < 1 day (8 counties). The populations of exposed and referent counties are similar with respect to age structure, ethnicity, population density, and socioeconomic status. Counties in eastern North Carolina are more rural and agricultural, with a higher percentage of African Americans, and of lower socioeconomic status than most of the remaining North Carolina counties. Demographic characteristics of the two groups of counties are available in Supplemental Material, Table 1 (http://dx.doi.org/10.1289/ehp.1003206).

We applied a Poisson regression model to daily counts of ED visits for combined and individual cardiovascular and respiratory outcomes separately, with explanatory variables indicating days within the 3-day window of dense smoke and subsequent 5 days of lagged exposure. The RR associated with days in which a county was exposed to wildfire smoke (and corresponding 5 lag days) was compared with other days in which that county was not exposed to smoke. For nonexposed (referent) counties, RR was calculated using the same days as were used for the exposed counties. RR was estimated separately for exposed and referent counties. The effects of exposure on outcomes at lags was estimated using an unconstrained distributed lag model (Peng and Dominici 2008). Previous studies (Braga et al. 2001; Pope et al. 2008) have determined that air pollution produces immediate and delayed effects on morbidity and mortality and that the time to adverse outcome may vary by pollutant and health outcomes. From the perspective of public health, in the present study we were interested in the total burden on human health associated with the wildfire episode. Inference on delay between the exposure and effect is not appropriate for this study without personal exposure measurements. Here, the results are reported in terms of the cumulative relative risk (cRR), that is, cumulative risk over lag days 0–5 after exposure (Peng and Dominici 2008) according to


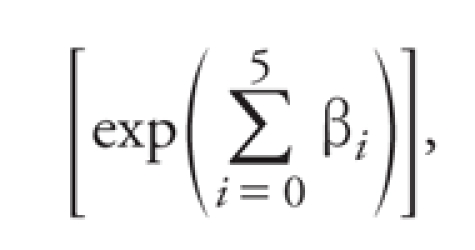
, [1]

where β*_i_* is the log RR estimate associated with the *i*th day after the exposure. Results are summarized in figures as the percent change in cRR or excess risk according to (cRR – 1) × 100% and as cRR in Supplemental Material, Tables 2–4 (http://dx.doi.org/10.1289/ehp.1003206). The analysis was stratified by age and sex in both exposed and referent county cohorts.

## Results

Asthma-related visits accounted for 44% of all respiratory codes considered, and heart failure accounted for 33% of all cardiac events. Consistent with the distribution of asthma prevalence by sex, the aggregate counts of asthma-related visits occurred in more women (70%) than men and in more individuals between 19 and 64 years (85%) compared with those ≥ 65 years of age. Cardiac events were substantially more common in individuals ≥ 65 years (67%) compared with those 45–64 years of age. The number of clinical events reported for each ICD-9-CM code during the study period is given in Table 1.

**Figure 3:**
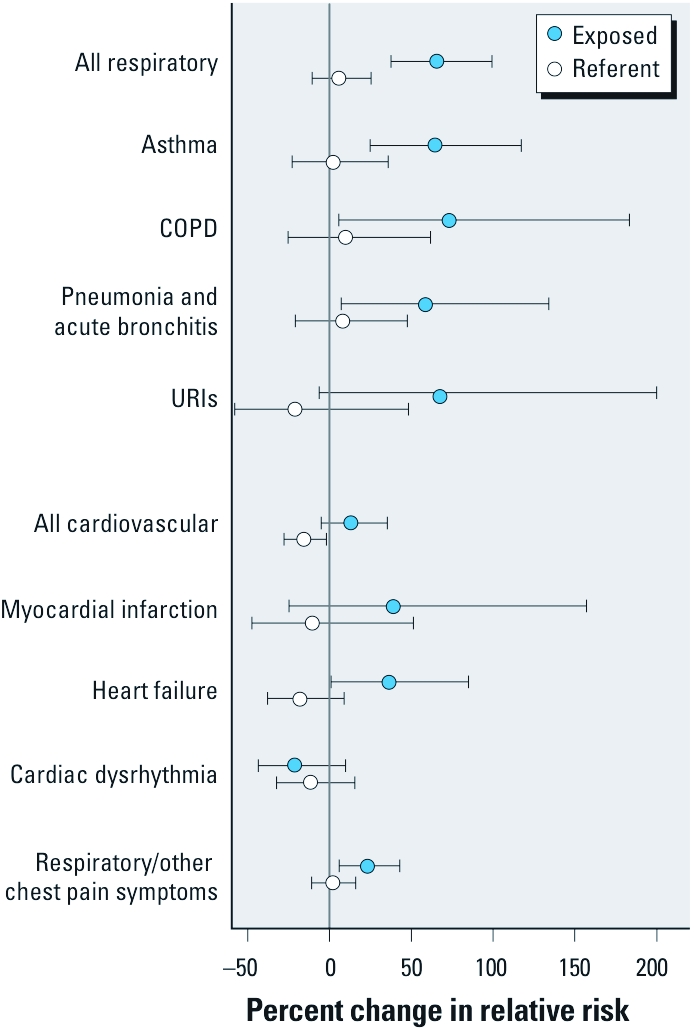
Percent change in cRR and 95% CIs by discharge diagnosis category for exposed and referent counties in North Carolina during the 3‑day period of high exposure compared with the entire 6‑week study period. The vertical gray line indicates the null hypothesis of no change in cRR.

In the exposed counties, ED visits for several outcomes were significantly increased during the high-exposure days and subsequent lag days compared with visits during the remainder of the 6-week study period in the exposed counties (Figure 3). ED visits for all the respiratory diagnoses were elevated in the exposed counties [cRR = 1.66; 95% confidence interval (CI), 1.38–1.99] but not in the referent counties [1.06 (0.89–1.25)]. Among the respiratory outcomes, ED visits for asthma [1.65 (1.25–2.17)], COPD [1.73 (1.06–2.83)], and pneumonia and acute bronchitis [1.59 (1.07–2.34)] increased significantly. Visits for URIs [1.68 (0.94–3.00)] also increased but were not statistically significant. We found no significant differences in respiratory outcomes when cumulative risks over the same calendar days were estimated for the referent counties.

In the exposed counties, cRR for heart failure–related ED visits [cRR = 1.37 (95% CI, 1.01–1.85)] during the 3 high-exposure days and 5 subsequent lag days was increased compared with other days, whereas visits for myocardial infarction and cardiac dysrhythmias were not increased for the same time periods in exposed or referent counties. Reflecting the increase in cardiac and respiratory events, ED visits associated with cardiopulmonary symptoms (ICD-9-CM code 786) were significantly increased [1.23 (1.06–1.43)] in the exposed counties.

**Figure 4:**
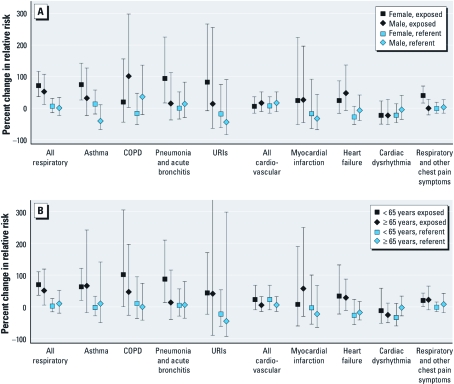
Percent change in cRR and 95% CIs by discharge diagnosis category for exposed and referent counties in North Carolina during the 3‑day period of high exposure compared with the entire 6‑week study period (*A*) by sex and (*B*) by age group. The 95% CI for URIs extending out of the figure reaches 1,816% in excess risk (cRR = 19.16) [see Supplemental Material, Table 4 (http://dx.doi.org/10.1289/ehp.1003206)].

Associations with respiratory outcomes varied by age and sex, and stratified analyses reflected higher uncertainty because of lower counts of events in these subgroups. In exposed counties, visits for asthma, pneumonia, acute bronchitis, and URI increased to a greater extent among women than among men (Figure 4A). In contrast, visits related to COPD were elevated only in men in exposed counties. There was a greater increase in ED visits for asthma, COPD, pneumonia, and acute bronchitis during exposed versus unexposed days among individuals < 65 years of age compared with those ≥ 65 years in the exposed counties (Figure 4B). We observed no differences in ED visits for cardiovascular events stratified by age or sex, possibly because the smaller number of visits for these ICD-9-CM codes diminished the power to observe effects in these subgroups.

## Discussion

This is the first population-based health study of peat bog fire exposures using a syndromic surveillance system with a nearly comprehensive record of health outcomes from an entire geographic region. We estimated the cRR of ED visits for cardiorespiratory outcomes associated with 3 high-exposure days and their corresponding 5 lag days. The study demonstrates that exposure to smoke from the wildfire increased ED visits for asthma, COPD, pneumonia, acute bronchitis, and heart failure in a sparsely populated nonurban area. The study also demonstrates the utility of syndromic surveillance in assessment of health burden during widespread environmental events. In turn, such assessments should help guide development of strategies and the allocation of resources for the public health response.

Consistent with the results from other studies, asthma-related outcomes were most prevalent, especially among adult women. A surprising and important observation is the statistically significant association between smoke exposure and an increase in ED visits for heart failure, as well as the trend toward a positive association with acute coronary syndrome (myocardial infarction and unstable angina). We restricted the cardiovascular clinical end points to acute coronary syndrome, which included myocardial infarction and unstable angina (ICD-9 410 and 411.1) and heart failure (ICD-9 428) and excluded hypertensive heart disease. Although previous studies have shown positive associations between ambient concentrations of PM_2.5_ and ED visits and hospitalization for heart failure (Brook et al. 2010; Dominici et al. 2007; Wellenius et al. 2005, 2006), to our knowledge this is the first study that has reported ED visits for heart failure associated with wildfire exposure. However, epidemiologic studies in areas with high residential wood burning have suggested that an increased risk of cardiovascular mortality and morbidity may be due, in part, to wood smoke emissions (Sanhueza et al. 2009; Schwartz et al. 1993). Our study demonstrated an increased percent change in RR of 37%. Compared with the rest of the state, counties of eastern North Carolina are among the poorest and least healthy counties, characterized by higher prevalence of hypertension, diabetes, ischemic heart disease, and heart failure (North Carolina State Center for Health Statistics 2009). These are clinical conditions that are associated with individuals more vulnerable to the health effects of ambient air particle pollution and may have contributed to the large number of events in this relatively sparse population (O’Neill et al. 2005, 2007; Wellenius et al. 2005).

Unlike the hot canopy forest fires often seen in the western portion of the country, the Pocosin fire was not associated with high temperatures and heat waves. Instead, a prolonged drought led to the unusually dry conditions in the region that allowed for rapid spread of fire across the peat bogs after a lightning strike. Therefore, it is not surprising that we did not observe long-term linear trends or seasonality for the principal respiratory and cardiac diagnoses during this study period as shown for visits for asthma (Figure 2). However, at least three types of misclassification in exposure could have occurred in the study. First, any days with elevated AOD levels outside the 3-day window were classified as unexposed days, which could have resulted in a bias toward the null hypothesis. Second, some of the referent counties were exposed to emissions from the fire at some point during the 6-week period, although not to the extent of the exposed counties, also potentially resulting in bias toward the null hypothesis. Finally, some misclassification at the individual level may have occurred because of differences in exposure between county groups. According to information from the 2000 census (U.S. Census Bureau 2010), exposed counties are demographically similar to but more rural than referent counties, which may have resulted in a higher exposure [see Supplemental Material, Table 1 (http://dx.doi.org/10.1289/ehp.1003206)]. However, no individual data were available for the analysis. Well-documented risks of increased morbidity and mortality due to air pollution have been based largely on central site monitoring and time-series studies of health outcomes within major urban centers. Rural and remote areas are often not studied because of sparse population and lack of monitoring data. The type and availability of monitoring most often found in sparsely populated areas, such as in this study, provide limited information about the geographic scope of exposure times of environmental events. Air pollution monitors collect data at irregular temporal resolution (e.g., every 24 hr, every 3 days, every 6 days) and do not provide comprehensive information regarding the geographic scope of the exposure, depending on monitor location, wind direction, and terrain (e.g., differences in elevation). Developing new methods for assessing exposure will be increasingly more important as wildfires and other environmental events become more frequent. Several computer models for atmospheric pollutant dispersion already exist but are difficult to validate against sparse ground measurements. In this study we used satellite-derived measurements of AOD to define spatial boundaries of the smoke plume. This allowed us to capture the geographic extent of ED visits in the region and thereby increase statistical power. Such exposure assessment, however, allows us to associate changes in RR to the exposure period but not to the concentration of air pollutants.

Central reporting of ED visits and the high rate of case identification afforded by the NC DETECT syndromic surveillance system contributed to the strength of the associations we found in this study. Syndromic surveillance systems were developed to detect epidemics and monitor outbreaks in near real-time (Buehler et al. 2008). The program is implemented at the state level; the median rate of hospital participation in the United States is 35% and ranges between 2 and 100%. Most states report chief complaint data alone, but a few, including North Carolina, report diagnostic ICD-9-CM codes. North Carolina has a uniquely comprehensive program, with 98% of its hospitals participating. Two other studies of health effects of wildfires in California and Florida (Johnson et al. 2005; Sorenset et al. 1999) used syndromic surveillance data and reported increases in respiratory outcomes, asthma, respiratory complaints, eye irritation, and smoke inhalation for respiratory-related chief complaints. The strength of evidence found in the present study further supports efforts to expand automated surveillance system for near-real-time delivery of information and health advisories during emergencies. Although ICD-9-CM codes are not as timely as chief complaints, they can provide more specific measurements of health outcomes and should be added to syndromic surveillance systems in other states.

Peat fires burn significantly more biological mass, produce massive amounts of smoke, and are notoriously more difficult to extinguish than hot canopy forest fires or grassland fires (Muraleedharan et al. 2000; Page et al. 2002; See et al. 2007; Soja et al. 2004). One study of peat fire particle composition reported carbonaceous particles, particularly organic carbon, nitrate, and sulfate as major components of PM_2.5_, whereas the less-abundant constituents included ions such as ammonium, nitrite, sodium, potassium; organic acids; and metals such as aluminum, iron, and titanium (See et al. 2007). In another study, Muraleedharan et al. (2000) reported carbon monoxide, CO_2_, and methane to be the most abundant gaseous emissions. Although emissions from peat fires may differ from those from forest fires in chemical composition, it is not known if they differ in toxicity.

Population growth and land use alterations are the primary bases for increased wildfire events worldwide. Additional stress is created by earlier snow melts, rising temperatures, cumulative effects of the current drought, and other climate-related changes (National Interagency Fire Center 2009; Westerling et al. 2006). Recent peat fires around Elektrogorsk, Russia, exemplify the synergistic effect of these factors and their impact on health and economy in the region (Reilly 2010). Unprecedented hot and dry weather in Russia during the summer of 2010 eased the spread of fires in swamps long ago drained to harvest energy from peat. The smoke obstructed ground and air travel and resulted in numerous health advisories (Williams 2010). Peat bogs have been exploited for energy use in many parts of the world, leaving vest areas of dried wetlands and swamps particularly vulnerable to droughts. Therefore, the risk of peat fires is likely to increase in upcoming decades (National Interagency Fire Center 2009).

## Conclusion

The consistent increase in RR in the exposed counties for nearly all outcome categories during and up to 5 days after exposure to wildfire smoke is striking and persuasive and has potentially significant public health implications. The precision of RR estimates in this study is attributed to the use of comprehensive population health data from the North Carolina public surveillance program as well as to the use of spatially and temporally dense satellite measurements of AOD. The region of the state most affected by the smoke was sparsely populated, with few available air quality monitors. Therefore, traditional exposure assessment based on monitoring and hospital admissions data alone would not provide us with enough information to assess the true risk. Both data sources used in this study are readily available to state and local public health officials and lend themselves to the application of traditional statistical methods. In the near future, public health officials may benefit from dashboard-like visualization tools that allow end users to overlay environmental data with health care data to improve event characterization capabilities and response efforts. Mitigation of exposure and raising public awareness would be expected to decrease the burden to the health care system and improve the well-being of the public.

## Supplemental Material

(112 KB) PDF

## References

[r1] Al-Saadi J, Szykman J, Pierce RB, Kittaka C, Neil D, Chu DA (2005). Improving national air quality forecasts with satellite aerosol observations.. Bull Am Meteorol Soc.

[r2] Braga AL, Zanobetti A, Schwartz J (2001). The lag structure between particulate air pollution and respiratory and cardiovascular deaths in 10 US cities.. J Occup Environ Med.

[r3] Brook RD, Rajagopalan S, Pope CA, Brook JR, Bhatnagar A, Diez-Roux AV (2010). Particulate matter air pollution and cardiovascular disease: an update to the scientific statement from the American Heart Association.. Circulation.

[r4] Buehler JW, Sonricker A, Paladin M, Soper P, Mostashari F (2008). Syndromic Surveillance Practice in the United States: Findings from a Survey of State, Territorial, and Selected Local Health Departments. Adv Dis Surveill 6(3):1–20.. http://www.syndromic.org/ADS/prepub/Buehler20080503.pdf.

[r5] Delfino RJ, Brummel S, Wu J, Stern H, Ostro B, Lipsett M (2009). The relationship of respiratory and cardiovascular hospital admissions to the southern California wildfires of 2003.. Occup Environ Med.

[r6] Dominici F, Peng RD, Zeger SL, White RH, Samet JM (2007). Particulate air pollution and mortality in the United States: did the risks change from 1987 to 2000?. Am J Epidemiol.

[r7] Duclos P, Sanderson LM, Lipset M (1990). The 1987 forest fire disaster in California: assessment of emergency room visits.. Arch Environ Health.

[r8] Engel-Cox JA, Holloman CH, Coutant BW, Hoff RM (2004). Qualitative and quantitative evaluation of MODIS satellite sensor data for regional and urban scale air quality.. Atmos Environ.

[r9] Johnson JM, Hicks L, McClean C, Ginsberg M (2005). Leveraging syndromic surveillance during the San Diego wildfires, 2003. MMWR Morb Mortal Wkly Rep.

[r10] Kunii O, Kanagawa S, Yajima I, Hisamatsu Y, Yamamura S, Amagai T (2002). The 1997 haze disaster in Indonesia: its air quality and health effects.. Arch Environ Health.

[r11] Künzli N, Avol E, Wu J, Gauderman WJ, Rappaport E, Millstein J (2006). Health effects of the 2003 Southern California wildfires on children.. Am J Respir Crit Care Med.

[r12] Moore D, Copes R, Fisk R, Joy R, Chan K, Brauer M. (2006). Population health effects of air quality changes due to forest fires in British Columbia in 2003: estimates from physician-visit billing data.. Can J Public Health.

[r13] Mott J, Mannino DM, Alverson CJ, Kiyu A, Hashim J, Lee T (2005). Cardiorespiratory hospitalizations associated with smoke exposure during the 1997, Southeast Asian forest fires.. Int J Hyg Environ Health.

[r14] Muraleedharan TR, Radojevic M, Waugh A, Caruana A (2000). Emissions from the combustion of peat: an experimental study.. Atmos Environ.

[r15] Naeher LP, Brauer M, Lipsett M, Zelikoff JT, Simpson CD, Koenig JQ (2007). Woodsmoke health effects: a review.. Inhal Toxicol.

[r16] National Interagency Fire Center (2009). Quadrennial Fire Review 2009. Final Report.. http://www.nifc.gov/PUBLICATIONS/QFR/QFR2009Final.pdf.

[r17] NC DETECT (2010). http://www.ncdetect.org/.

[r18] North Carolina State Center for Health Statistics (2009). 2009 BRFSS Topics for Eastern North Carolina.. http://www.schs.state.nc.us/SCHS/brfss/2009/east/topics.html.

[r19] O’Neill MS, Veves A, Sarnat JA, Zanobetti A, Gold DR, Economides PA (2007). Air pollution and inflammation in type 2 diabetes: a mechanism for susceptibility.. Occup Environ Med.

[r20] O’Neill MS, Veves A, Zanobetti A, Sarnat JA, Gold DR, Economides PA (2005). Diabetes enhances vulnerability to particulate air pollution-associated impairment in vascular reactivity and endothelial function.. Circulation.

[r21] Page S, Siegert F, Rieley J, Boehm H, Jaya A, Limin S. (2002). The amount of carbon released from peat and forest fires in Indonesia during 1997.. Nature.

[r22] Peng D Dominici F 2008 Statistical Methods for Environmental Epidemiology with R. New York:Springer Science + Business Media, LLC.

[r23] Pope CA, Renlund DG, Kfoury AG, May HT, Horne BD (2008). Relation of heart failure hospitalization to exposure to fine particulate air pollution.. Am J Cardiol.

[r24] Reilly M (2010). Russian fires, heat waves, and a drive through hell.. http://news.discovery.com.

[r25] Sanhueza PA, Torreblanca MA, Diaz-Robles LA, Schiappacasse LN, Silva MP, Astete TD (2009). Particulate air pollution and health effects for cardiovascular and respiratory causes in Temuco, Chile: a wood-smoke-polluted urban area.. J Air Waste Manag Assoc.

[r26] Sastry N. (2002). Forest fires, air pollution, and mortality in Southeast Asia.. Demography.

[r27] Schwartz J, Slater D, Larson TV, Pierson WE, Koenig JQ (1993). Particulate air pollution and hospital emergency room visits for asthma in Seattle.. Am Rev Respir Dis.

[r28] See S, Balasubramanian R, Rianawati E, Karthikevyan S, Streets D. (2007). Characterization and source apportionment of particulate matter ≤ 2.5 µm in Sumatra, Indonesia, during a recent peat fire episode.. Environ Sci Technol.

[r29] Soja AJ Cofer WR Shugart HH Sukhinin AI Stackhouse PW McRae DJ 2004 Estimating fire emissions and disparities in boreal Siberia (1998–2002). J Geophys Res Atmos 109 D14S06; doi:10.1029/2004JD004570 [Online 23 July 2004]

[r30] Sorenset B, Fuss M, Mulla Z, Bigler W, Wiersma S, Hopkins R. (1999). Surveillance of morbidity during wildfires—Central Florida, 1998.. MMWR Morb Mortal Wkly Rep.

[r31] U.S. Census Bureau (2010). State & County QuickFacts.. http://quickfacts.census.gov/qfd/index.html.

[r32] U.S. EPA (U.S. Environmental Protection Agency) (2009a).

[r33] U.S. EPA (U.S. Environmental Protection Agency) (2009b). Integrated Science Assessment for Particulate Matter. EPA/600/R-08/139F.

[r34] U.S. Public Health Service and Health Care Financing Administration (1980). International Statistical Classification of Diseases, Injuries, and Causes of Death. Ninth Revision. Clinical Modification. DHHS No. (PHS) 80-1260.

[r35] Vedal S, Dutton SJ (2006). Wildfire air pollution and daily mortality in a large urban area.. Environ Res.

[r36] Wang J Christopher SA 2003 Intercomparison between satellite-derived aerosol optical thickness and PM_2.5_ mass: implications for air quality studies. Geophys Res Lett 30 21); doi:10.1029/2003GL018174 [Online 6 November 2003]

[r37] Wellenius GA, Bateson TF, Mittleman MA, Schwartz J (2005). Particulate air pollution and the rate of hospitalization for congestive heart failure among medicare beneficiaries in Pittsburgh, Pennsylvania.. Am J Epidemiol.

[r38] Wellenius GA, Schwartz J, Mittleman MA (2006). Particulate air pollution and hospital admissions for congestive heart failure in seven United States cities.. Am J Cardiol.

[r39] Westerling AL, Hidalgo HG, Cayan DR, Swetnam TW (2006). Warming and earlier spring increase western US forest wildfire activity.. Science.

[r40] Williams S (2010). Smog from Fires Chokes Moscow.. http://news.discovery.com/earth/moscow-smog-wildfires-russia.html.

