# Supplementary material for: Peat Bog Wildfire Smoke Exposure in Rural North Carolina Is Associated with Cardiopulmonary Emergency Department Visits Assessed through Syndromic Surveillance
Source: Environ Health Perspect. 2011 Oct 1;119(10):1415–20. doi: 10.1289/ehp.1003206 (PMC3230437; doi:10.1289/ehp.1003206)
Supplement: (112 KB) PDF [file ehp.1003206.s001.pdf]

Supplemental Material

The Use of Syndromic Surveillance to Identify an Association between Cardio-Pulmonary Emergency Department Visits and Wildfire Smoke Exposure

Ana G. Rappold, Susan L. Stone, Wayne E. Cascio, Lucas M. Neas, Vasu J. Kilaru, Martha Sue Carraway, James J. Szykman, Amy Ising, William E. Cleve, John T. Meredith, Heather Vaughan-Batten, Lana Deyneka, and Robert B. Devlin

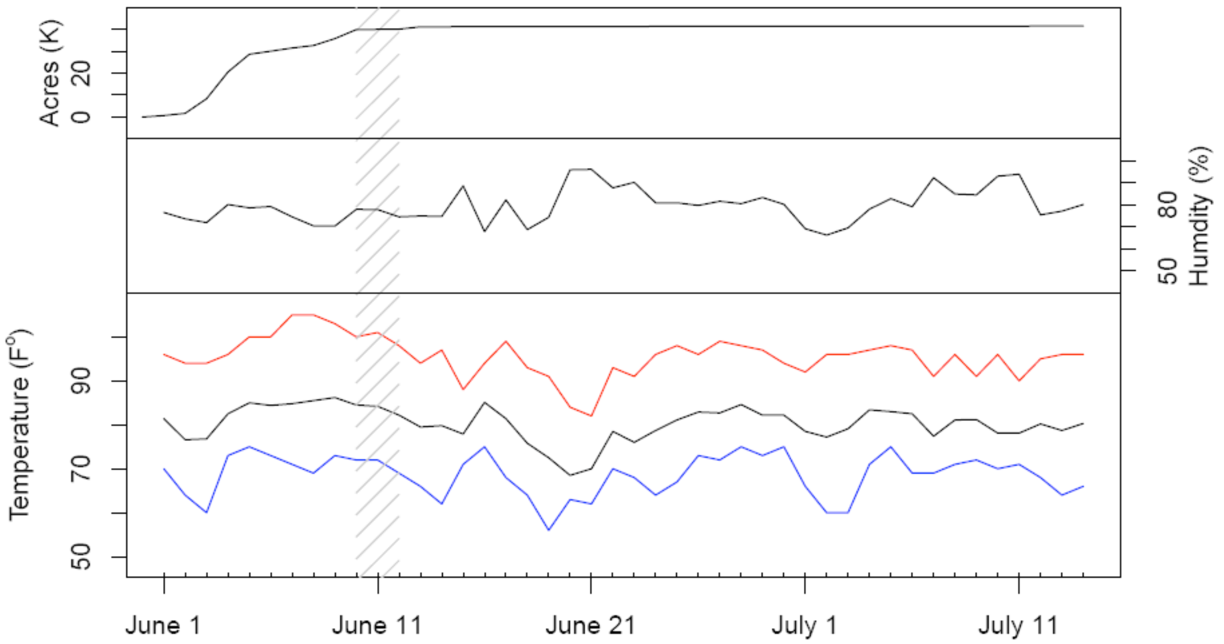

Supplemental Material, Figure 1
The figure illustrates progression of fire and daily meteorological conditions during the study period (June 1st -July 14th). Bottom panel gives daily minimum (blue), mean (black), and maximum (red) temperatures in degrees Fahrenheit. Middle panel gives relative humidity as percent while top panel give cumulative account of burned acreage (in thousands) during the fire. The three day episode of high exposure June 10th -12th is marked with dashed grey lines.

	North Carolina	Exposed Counties	Referent Counties
Population, 2000	8,046,500	891,194	1,528,057
Population, 2008	9,222,414	991,040	1,679,052
Percent change 2000 to 2008	+14.6	+11.0	+12.5
Geographic area (square miles)	48,711	9,339	13,204
Population density, 2008	189.3	106.1	127.2
Population characteristics in 2000			
White (%)	73.9	65.0	61.0
Black (%)	21.6	29.7	30.3
Hispanic (%)	7.4	5.4	4.2
Males (%)	49.0	48.9	49.5
Under 18 years of age (%)	24.4	24.9	25.6
18 to 64 years of age (%)	63.6	62.6	62.9
65 years of age and over (%)	12.0	12.5	11.5
Below poverty in 2007 (%)	14.6	17.4	17.6
Per capita income in 1999	$20,307	$17,297	$17,121
Housing characteristics in 2000			
Average household size	2.57	2.60	2.66
Owner occupied housing (%)	69.4	68.2	68.5
Same house in 1995 (%)	53.0	54.1	53.1
Land use characteristics			
Total farms in 1997	49,406	7,674	8,328
Crop acreage in 1997 (%)	18.0	27.9	20.0

Supplemental Material, Table 1
Population, housing, and land use characteristics of North Carolina as a whole and of exposed and referent counties (source US Census 2000).

	Exposed Counties			Referent Counties		
	RR	2.50%	97.50%	RR	2.50%	97.50%
All Respiratory	1.66	1.38	1.99	1.06	0.89	1.25
Asthma	1.65	1.25	2.17	1.02	0.77	1.36
COPD	1.73	1.06	2.83	1.1	0.75	1.62
Pneumonia & Acute Bronchitis	1.59	1.07	2.34	1.08	0.79	1.47
Upper Respiratory Infections	1.68	0.94	3	0.79	0.42	1.49
All Cardiovascular	1.13	0.95	1.35	0.84	0.72	0.98
Myocardial Infarction	1.39	0.75	2.57	0.9	0.53	1.51
Heart Failure	1.37	1.01	1.85	0.82	0.62	1.09
Cardiac Dysrhythmia	0.79	0.57	1.1	0.88	0.68	1.15
Respiratory/ Other Chest Pain Symptoms	1.23	1.06	1.43	1.02	0.89	1.16

Supplemental Material, Table 2
Estimates of cumulative relative risk (RR) and 95% confidence intervals by discharge diagnosis category for exposed and referent North Carolina counties.

	Exposed Counties						Referent Counties					
	Female			Male			Female			Male		
	RR	2.5%	97.5%	RR	2.5%	97.5%	RR	2.5%	97.5%	RR	2.5%	97.5%
All Respiratory	1.72	1.36	2.17	1.53	1.12	2.07	1.06	0.86	1.31	1.01	0.76	1.34
Asthma	1.75	1.26	2.42	1.32	0.77	2.27	1.14	0.82	1.58	0.6	0.32	1.11
COPD	1.2	0.56	2.56	2.02	1.02	3.98	0.84	0.48	1.47	1.37	0.8	2.36
Pneumonia & Acute Bronchitis	1.94	1.16	3.25	1.15	0.63	2.12	1	0.66	1.52	1.14	0.71	1.82
Upper Respiratory Infections	1.83	0.92	3.67	1.14	0.36	3.56	0.82	0.38	1.75	0.56	0.16	1.9
All Cardiovascular	1.07	0.84	1.36	1.17	0.9	1.52	1.07	0.84	1.36	1.17	0.9	1.52
Myocardial Infarction	1.25	0.48	3.23	1.27	0.54	2.96	0.83	0.36	1.92	0.67	0.32	1.43
Heart Failure	1.25	0.84	1.87	1.48	0.93	2.37	0.72	0.49	1.06	0.93	0.61	1.41
Cardiac Dysrhythmia	0.78	0.5	1.23	0.79	0.48	1.28	0.79	0.54	1.14	0.95	0.65	1.41
Respiratory/ Other Chest Pain Symptoms	1.4	1.16	1.7	1	0.79	1.27	1	0.84	1.19	1.03	0.84	1.27

Supplemental Material, Table 3
Cumulative relative risk and 95% confidence intervals by discharge diagnosis category and gender for exposed and referent North Carolina counties.

	Exposed Counties						Referent Counties					
	<65			≥65			<65			≥65		
	RR	2.5%	97.5%	RR	2.5%	97.5%	RR	2.5%	97.5%	RR	2.5%	97.5%
All Respiratory	1.7	1.37	2.1	1.52	1.06	2.19	1.03	0.85	1.26	1.11	0.81	1.52
Asthma	1.64	1.21	2.21	1.67	0.82	3.42	0.98	0.72	1.34	1.11	0.51	2.41
COPD	2.02	1	4.05	1.48	0.74	2.97	1.11	0.64	1.95	1.01	0.58	1.74
Pneumonia & Acute Bronchitis	1.87	1.13	3.1	1.15	0.61	2.16	1.06	0.71	1.56	1.08	0.65	1.8
Upper Respiratory Infections	1.44	0.77	2.71	1.43	0.11	19.16	0.78	0.39	1.54	0.55	0.08	3.98
All Cardiovascular	1.24	0.91	1.68	1.06	0.85	1.32	1.24	0.91	1.68	1.06	0.85	1.32
Myocardial Infarction	1.08	0.4	2.89	1.58	0.71	3.5	0.96	0.46	2	0.78	0.36	1.67
Heart Failure	1.34	0.78	2.32	1.29	0.89	1.87	0.74	0.44	1.24	0.83	0.59	1.17
Cardiac Dysrhythmia	0.88	0.49	1.59	0.75	0.5	1.12	0.67	0.41	1.11	0.98	0.72	1.34
Respiratory/ Other Chest Pain Symptoms	1.21	1.01	1.43	1.23	0.91	1.65	0.99	0.85	1.15	1.08	0.82	1.43

Supplemental Material, Table 4

Cumulative relative risk and 95% confidence intervals by discharge diagnosis category and age group for exposed and referent North Carolina counties.
